# Longevity as a matter of housekeeping

**DOI:** 10.18632/aging.100173

**Published:** 2010-07-16

**Authors:** Troy A. A. Harkness

**Affiliations:** Department of Anatomy and Cell Biology, University of Saskatchewan, Saskatoon, SK, S7N 5E5, Canada

Despite intense investigation into the causes of aging, molecular mechanisms governing cellular longevity remain elusive, yet some possibilities are coming into focus [1,2]. It has become clear from many studies utilizing simple model systems that the aging process is multifarious and certainly involves a combination of genetic and stochastic mechanisms. The discovery of single gene mutations that influence lifespan has provided strong support for a genetic component driving cellular longevity [3]. The switch between stress and nutrient availability appears to play a central role in lifespan. The conserved insulin-signaling pathway has drawn a significant amount of attention over the past few years as a major lifespan determining signaling network. In many systems, impairing this pathway impedes the ability of caloric restriction (CR) to enhance lifespan, suggesting that nutrient sensing is key to CR.

In this issue of Aging, the Titorenko laboratory (Goldberg et al.) tested the hypothesis that networks exist within cells that are not inducible, but act constitutively to extend the lifespan of cells regardless of nutrient availability [4]. To test their hypothesis they used a chemical genetic approach that took advantage of the pex5∆ mutation that impairs peroxisomal import and hinders free fatty acid (FFA) oxidation. FFA's are important, as they are used to generate acetyl-CoA, a requirement for ATP synthesis during CR. As predicted, pex5∆ cells exhibited a much shorter lifespan under CR (0.5% glucose) compared to normal laboratory conditions (2% glucose). Therefore, under CR conditions, ~19,000 small molecules were screened for their ability to extend pex5∆ lifespan. Of the 24 positives identified, the highly hydrophobic lithocholic acid (LCA) was the most effective at increasing pex5∆ CR lifespan. This result provided evidence that a housekeeping longevity assurance mechanism was in place that was independent of CR, and that LCA was capable of activating it. If this is true, then lifespan of wild type cells under normal and CR conditions should also be influenced by LCA. This was indeed the case as demonstrated in Goldberg et al.

Next, the Titorenko group tested the Tor1 and Ras2 nutrient sensing pathways for their lifespan response to LCA. While it was found that the extended lifespan of tor1∆ and ras2∆ could be extended by LCA, the effect was more pronounced in tor1∆ cells. The authors concluded that LCA extended lifespan via modulation of a pathway that does not overlap with the major nutrient sensing pathways involving Tor1 and Ras2. The limited sensitivity of ras2∆ cells to LCA prompted the authors to suggest that LCA extends lifespan under non-CR conditions by unmasking a previously unknown role for the Ras/PKA pathway in extending lifespan. This is a novel and very exciting study that sheds light on previously unidentified mechanisms employed to increase lifespan in model systems. Many new avenues of study are now available, such as understanding the potential dual role of Ras/PKA in lifespan determination, and what influence LCA may have on higher eukaryotic systems. The many cellular processes affected by LCA underscores the fact that aging is far more complex than originally anticipated. Nonetheless, the identification of simple small molecules with such an impact on lifespan is a large step forward in our endeavor to understand the aging process, and perhaps, how to manipulate it.
